# Correction to “Psychological and motivational factors associated with addiction tendencies to gaming, video viewing, and social networking services use among high school students: a cross‐sectional study”

**DOI:** 10.1002/pcn5.70303

**Published:** 2026-02-16

Inaguma T, Funatogawa T, Sekizaki R, Nakamura Y, Mizuno M, Nemoto T. Psychological and motivational factors associated with addiction tendencies to gaming, video viewing, and social networking services use among high school students: a cross‐sectional study. Psychiatry Clin Neurosci Rep. 2025; 4:e70218. https://doi.org/10.1002/pcn5.70218

In the Note of Table 2, the text “Variables with *p* > 0.05 are marked with an asterisk.” was incorrect. This should have read: “Variables with *p* < 0.05 are marked with an asterisk.”

We apologize for this error.

